# Practice and Intention to use long acting and permanent contraceptive methods among married women in Ethiopia: Systematic meta-analysis

**DOI:** 10.1186/s12978-016-0194-0

**Published:** 2016-06-21

**Authors:** Yonatan Moges Mesfin, Kelemu Tilahun Kibret

**Affiliations:** Department of Public Health, College of Medical and Health Science, Haramaya University, Harar, Ethiopia; Department of Public Health, College of Medical and Health Science, Wollega University, Nekemte, Ethiopia

**Keywords:** LAPCMs, Intention, Practice, Meta-analysis, Systematic review, Ethiopia

## Abstract

**Background:**

The long acting and permanent contraceptive methods (LAPCMs) has not used unlike that of short-acting methods in Ethiopia. Ethiopia is the second most populous country in Sub Saharan Africa with a high total fertility rate, and high maternal and child mortality rates. This study summarized the evidence of practice and intention to use long acting and permanent family planning methods among women in Ethiopia using systemic review and meta-analysis.

**Methods:**

A systematic review and meta-analysis of the published and unpublished observational studies were conducted. Original studies were identified using databases of Medline/Pubmed, and Google Scholar. Heterogeneity across studies was checked using Cochrane Q test statistic and I^2^test. The pooled proportion of intention to use and the practice of long acting and permanent contraceptive methods were computed using a/the random effect model.

**Results:**

Based on the ten observational studies included in the meta-analysis, the pooled prevalence of intention to use long acting and permanent contraceptive methods among married women according to the random effect model was 42.98 % (95 % CI 32.53, 53.27 %). On the other hand, the pooled practice of long acting and permanent methods of contraceptive among the study participants was 16.64 % (95 % CI 12.4 to 20.87 %).

**Conclusion:**

This meta-analysis revealed that women’s intention to use LAPCMs is generally good but their utilization is low. It is recommended, therefore, that LAPMCs must be made more readily available and accessible to women at the lower level of health service delivery who are in need of it.

## Background

Family planning (FP) is a process that usually involves a discussion between a woman, a man, and a trained FP service provider focusing on family health and the desires of the couple to either limit or space their children [1]. Contraceptive methods used for FP can be grouped programmatically into two categories. These are long-acting and permanent contraceptive methods (intrauterine devices, implants, and sterilization) and short-term methods (pills, condoms, spermicides, injectables, other modern methods, and all traditional methods). Long-acting and permanent contraception methods (LAPCMs) are generally used to limit childbearing, whereas short acting methods are important for birth delay and birth spacing [2].

Researchers have shown that many women do not use oral contraceptives correctly and consistently during every act of sexual intercourse. As a result 1 million pregnancies occur from the faulty use of oral contraceptives each year and unintended pregnancies remain common. The method of contraception must be tailored to meet the needs of such individuals [3, 4]. If more effective LAPCMs were used, unintended births and induced abortions could be substantively reduced to help families and countries achieve their health goals [5]. Moreover, removing barriers to the use of contraceptives and enhancing the demand for family planning could avert 54 million unintended pregnancies, more than 79,000 maternal deaths and 1 million infant deaths throughout the world each year [6]. LAPCMs remain a relatively small and sometimes missing component of national FP programs in Sub Saharan Africa (SSA). These contraceptive methods have the potential to enhance FP programs in meaningful ways if the challenges to their availability, accessibility and acceptability can be overcome [3].

The total fertility rate of Ethiopia is 4.1 children per woman. A great majority of the health facilities in Ethiopia offer the oral contraceptive pill (98.8 %) and injectable contraception (98.0 %) followed by the male condom (95.2 %), an implant(75.0 %), an intra uterine device (IUD) (53.6 %), female sterilization (22.6 %), male sterilization (16.7 %), and the female condom (4.0 %) [6]. The modern contraceptive prevalence rate among currently married women in Ethiopia is 40 %. Thirty seven percent of married women want no more children but only 3.4 % of married women reported using implants, 0.3 % IUD, and less than 1 % are sterilized [7].

In a country like Ethiopia with a high fertility rate and an unmet need of contraceptives, shifting towards LAPCMs is an important strategy to ensure continuity of the services. But the issue is controversial; the contraceptive method mix is dominated by short-term methods like pills and injectables [8–11]. The Ethiopian Ministry of Health has planned and has been working on the provision of all FP methods, especially LAPCMs, in the lowest service delivery level [12]. Despite the fact that modern contraceptive services are made accessible nearly all major urban areas in Ethiopia and in most instances at lower or no cost but still not all women utilizing it [13].

Pocket studies in different part of Ethiopia were conducted to estimate the magnitude of practice and intention to use long acting and permanent contraceptive methods among married women. These studies showed that the intention to use long acting and permanent contraceptive methods among married women ranges from 27.3 % in Goba town, South East Ethiopia [8], to 65 % in Jinka town, Southern Ethiopia [14]. The practice of LAPCMs among married women also ranged from 7.3 % in Jinka town, Southern Ethiopia [14], to 19.5 % in Debre Markos town, North Ethiopia [15]. LAPCMs are one of the key elements for the reduction of child and maternal mortality directly, and for achieving other MDG goals indirectly.

It is important to have summarized evidence on the practice rate and intention to use LAPCMs among married women as this helps the concerned bodies to identify existing gaps and propose supplementary strategies to increase the availability, accessibility and utilization of LAPCMs in Ethiopia. Therefore, the aim of this study was to summarize the evidence of practice and intention to use long acting and permanent family planning methods among women in Ethiopia.

## Methods

### Study design and data source

This study was a systemic review and meta-analysis of the published and unpublished observational studies on prevalence rate of practice and intention to use LAPCMs among married women in Ethiopia. English language publications in the Medline data base, Google Scholar and HINARI (Health Inter Network Access to Research Initiative) were identified and cross-checked with reference lists containing combinations of the key words “intention *to use”* “*demand” and “prevalence rate of LAPCMs”*. In addition, a search was also made for cross-reference lists of identified original articles and reviews for other relevant articles. The data abstraction was performed from October 1 to June, 2015.

### Study selection

A systematic review and meta-analysis were made on cross-sectional studies that were focused on the intention to use and practice of LAPCMs among married women in Ethiopia. We included articles in the meta-analysis if they reported the practice and/or intention to use LAPCMs among married women in Ethiopia without restriction of publication date. Reports of original studies, unpublished master theses and PhD dissertations which are written in English language were also included. Studies were excluded from the analysis for any of the following reasons: articles were focused on short term contraceptives, meta-analysis or systematic reviews; articles consisted of comments, editorials, or duplicate publication of the same study; response rate was less than 80 %, articles available only in abstract form and articles with sample size of less than 50. The selection of articles for review was done in three stages: titles alone, abstracts, and then full-text articles.

### Methodological quality assessment

Study quality indicators were sample size, reporting of response rate and appraisal of external validity of the study. Studies were assessed for quality and those with high quality were included for analysis. High quality studies were studies that: reported outcomes on at least 50 patients; had response rates greater than 80 %; and, reported on either practice of LAPCMs and/or intention to use LAPCMs.

### Data abstraction

The data abstraction was conducted independently by two investigators (YM, KT). The selected studies were reviewed by using pretested and standardized abstraction format and the following data were extracted: title, authors, year of publication, study site/base (community-based or institution-based), sample size, response rates, and measure of rate with its confidence interval (CI). When there was a discrepancy in data abstraction between the investigators, it was resolved through discussion and consensus.

### Statistical analysis

STATA version 11.0 software was used for data entry and analysis. The descriptions of original studies were made using tables and forest plots. The overall effect (pooled estimated prevalence rate) of LAPCMs practice and intention to use was carried out by using a random effects model and measured by using a prevalence rate with 95 % confidence intervals [95 % CI]. Heterogeneity across studies was estimated by Cochran’s Q test [16] and I^2^test which shows the proportion of total variation across studies that is due to heterogeneity rather than to chance [17].

## Result

**Fig. 1:**
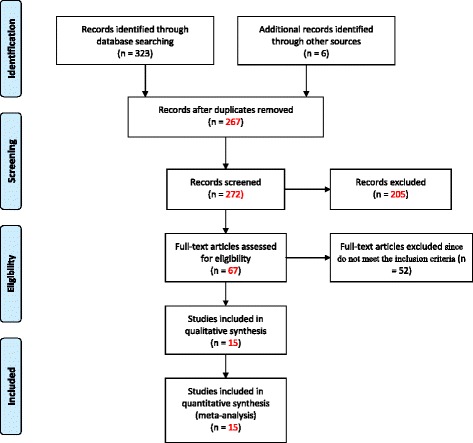
Flow chart diagram describing selection of studies for the systematic review and meta-analysis (identification, screening, eligible and included studies). Articles may have been excluded for more than one reason, 2015

A total of 328 articles were identified through the literature search. Four additional articles were also manually obtained. Of these 329 articles, 267 were excluded after screening by titles and abstracts. These were duplicated studies, case reports and reviews. Of the remaining 62 articles, 51 studies were excluded because 24 studies were conducted outside Ethiopia, ten studies dealt with short term contraceptive methods and 17 studies were based on qualitative findings. Finally, 16 articles were used for the meta-analysis with a total population of 18,569. Ten of the articles were relevant for intention to use LAPCMs and nine were relevant for practice. See Fig. 1 for the flow diagram for the study selection.

## Characteristics of studies included in the review

All of the 16 selected studies for the analysis were cross sectional studies [7, 8, 11, 14, 15, 18–20, 22–28] conducted between 2008 and 2015 with study populations varying from 203 to 10,204. All studies were reported in English. General characteristics and descriptions of the studies selected for meta- analysis are outlined in Table 1.

**Table 1:** Summary of the 16 observational studies included in the meta-analysis assessing women intention to use and practice of LAPCMs in Ethiopia, 2015

First author, year,	Study period	Study setting/base	Study population	Sample size	Parameter studied	Prevalence (%), Confidence interval
Gebremariam and Addissie [19], 2014	2012	Community based	Married women of reproductive age	594	Intention to use LAPCMs	48.4 (44.1 - 52.7)
Takele et al. [8], 2014	2009	Community based	Married women of reproductive age	734	Practice and intention to use LAPCMs	8.7 (6.9 - 11)
						27.3 (24.2 - 30.612)
Meskel and mekonene [18], 2014	2013	Health facility based	Women who were using STCs	416	Intention to use LAPCMs	38 (33.3 - 42.7)
Bulto et al. [15], 2014	2012	Community based	Married women of reproductive age	519	Practice and intention to use LAPCMs	19.5 (16.3 - 23.1)
						52.4 (48.1 - 56.7)
Getachew Mekonnen, [14] 2013	2008	Community based	Women of reproductive age group	800	Practice and intention to use LAPCMs	7.3 (5.7 - 9.3)
						65.2 (61.9 - 68.5)
Haile A, Fantahun M, [22] 2012		Community based		398	Intention to use LAPCMs	24.4 (20.4 - 28.9)
EDHS [21], 2011	2010	Community based	Married and sexually active unmarried women	10,204	Intention to use LAPMCs	54.0 (53.0 - 55.0)
Dashe Negawo, unpublished [20]	2010	Health facility based	Women who were using STCs	519	Intention to use LAPCMs	56.1 (51.8 - 60.3)
Alemayehu et al. [11], 2012	2012	Community based	Married women of reproductive age	460	Practice of LAPMCs	12.3 (9.6 - 15.6)
Tesfaledet T, [23] 2015	2014	Community based	Married women of reproductive age	802	Intention to use LAPCMs	18.2 (15.65 - 21)
Alemu S, [24] 2015	2014	Community based	Married women of reproductive age	1003	Practice of LAPCMs	20 (17.65 - 22.6)
Walelgn G et al., 2015 (unpublished) [25]	2015	Community based	Married women of reproductive age	662	Practice of LAPCMs	163 (13.5,19.1)
Haregewoyn K et al., 2015 (unpublished) [26]	2015	Community based	women of reproductive age	393	Practice of LAPCMs	25.2 (21.0,29.5)
Wanzahun G et al. 2015 [27]	2015	Health facility based	Women of reproductive age	203	Practice of LAPCMs	22.9 (17.1,28.7)
Amanuel A 2014 [28]	2014	Community based	Married women of reproductive age	343	Intention to use LAPCMs	45.9 (40.6,51.2)
Gizachew A, 2014	2013	Community based	Women of reproductive age	519	Practice LAPCMs	19.5 (16,22.9)

### Assessing heterogeneity

Ten studies dealing with intention to use LAPCMs showed high heterogeneity using the Cochrane Q test (Q test *p* = 0.0000) and I^2^ test (I^2^ = 99.1 %) which is indicative for random effects modeling. Nine studies which are included in this meta-analysis to pool the practice of the married women towards LAPCMs also showed high heterogeneity using Cochrane Q test (Q test *p* = 0.0000) and I^2^ test (I^2^ = 95.0 %).

## Meta-analysis results

### Intention to use long acting and permanent methods of contraception

Among the 15 studies eligible for meta-analysis, ten studies were focused on the intention of women to use LAPCMs. A study in Jinka in South Ethiopia revealed that nearly two thirds of participants had the intention to use LAPCMs [14]. Another study from North West Ethiopia in Debre Markos showed that 45.9 % of women had the intention to use one of the LAPCMs in the future. Of these, 98 (18.9 %) of the respondents intended to use Implanon, 67 (12.9 %) Jadele, and 60 (11.6 %) IUD [15]. A study in Wolayita, Southern Ethiopia, reported tha t 38 % of women had the intention to use LAPCMs [18].

According to a study conducted in Goba, South East Ethiopia, the total demand for LAPCMs in the town was 18.12 %. The unmet need for LAPMCs was 9.4 % (3.78 % for spacing children and 5.59 % for limiting children). The overall prevalence rate of intention to use LAPCMs in the study area was 27.3 % [8].

Another study conducted in Adigrat in the Tigray region, North Ethiopia, showed that the prevalence of intention to use LAPMCs was 48.4 % (95 % CI = 44.1, 52.7) while 14.6 % participants were unsure of their intention. Of those who had intention, 58.9 % had intention to use one of the LAPCMs within the next 1 year. The most preferred method participants ‘indicated an intention to use was implants (71.3 %), followed by IUCD (24.0 %). This study also demonstrated that intention to use LAPCMs was higher among women who knew at least one of LAPCMs (AOR = 4.7, 95 % CI = 1.58, 14.01) and women who do not want to have birth within the next 2 years (AOR = 1.9, 95 % CI = 1.22, 3.13). The intention to use LAPCMs was less among women who perceive poor support from their husbands (AOR = 0.2, 95 % CI = 0.09, 0.45) and those who perceive LAPCMs as harmful to the womb (AOR = 0.24, 95 % CI = 0.14, 0.41) [19].

A study conducted in Ambo, Ethiopia, in 2010 documented that 291 (56.1 %) of 519 women who were using family planning methods had the intention to use LAPCMs [20]. Likewise, results from the Ethiopian Demography and Health Survey in 2011 show that the magnitude rate of intention to use among the study population was 54 % [21]. On the other hand, the facility based study conducted in East Showa Zone in Batu, Ethiopia, in May 2009 showed that, among 398 women aged 18 – 49 years, the magnitude rate of intention to use LAPCMs was 24.4 % [22].

**Fig. 2:**
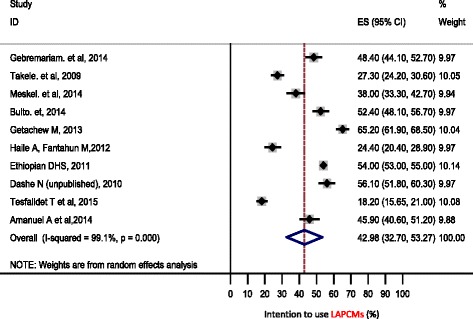
Forest Plot of the 10 Cross-Sectional Studies that quantitatively assessed the magnitude of intention to use long acting and permanent family planning methods among married women in the reproductive age group, 2015

Based on ten observational studies included in the meta-analysis, the pooled prevalence of intention to use LAPCMs among married women according to the random effect model was 42.98 % (95 % CI 32.53, 53.27 %) (Fig. 2).

### Practices of LAPCMs among women of reproductive age

Of the 15 studies identified, 8 studies were dealing with practice of women on LAPCMs. According to a study from Jinka, South Ethiopia, the prevalence of long acting and permanent contraceptive methods was 7.3 %. About one out of every five participants has ever received information about one or more of the long acting and permanent contraceptive methods. In addition, a study from Goba, South East Ethiopia, showed the utilization of LAPCMs in the town as 8.7 % and the unmet need for LAPCMs as 9.4 %. Moreover, according to a study report from Mekelle, North Ethiopia, the overall prevalence of long acting and permanent contraceptive methods was 12.3 %. Of those married women who were using LAPCMs, the majority of women (87 %) used implants followed by IUCD (13 %), and there were no married women who underwent female sterilization. Unlike the previous three studies, a study from Debre Markos Town, North West Ethiopia reported that 62.2 % of them were using modern family planning (FP) methods in which 19.5 % were using long acting and permanent contraceptive methods (LAPCMs).

**Fig. 3:**
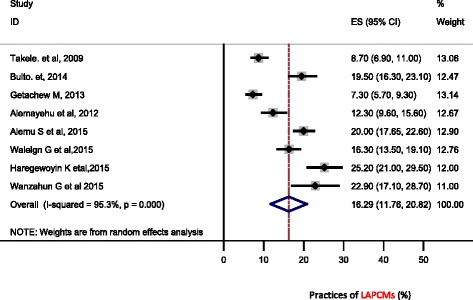
Forest Plot of the 8 Cross-Sectional Studies that quantitatively assessed the prevalence rate of long acting and permanent family planning methods practice among married women, 2015

Based on the nine studies included in this meta-analysis, the pooled practice prevalence rate of long acting and permanent contraceptive methods among women was 16.64 % (95 % CI 12.4 to 20.87 %) (Fig. 3).

## Discussion

The systemic review and meta-analysis targeted articles on the magnitude of utilization and intention to use (LAPCMs), with 15 studies selected. Fifteen studies were identified from regional states of Ethiopia and one national survey [7]. The evidence was summarized in terms of the magnitude of utilization and intention to use long acting and permanent family planning method among women in the reproductive age group. According to the results of this meta-analysis, the pooled magnitude of practice of and intention to use LAPCMs among women in the reproductive age group were 16.64 and 42.98 %, respectively.

Long-acting and permanent methods of contraception (LAPCMs) can address a full range of women’s and couples ‘family planning needs. According to the results of this meta-analysis, the pooled magnitude of intention to use LAPCMs among women in the reproductive age group was 42.66 %. In the individual studies, the intention to use LAPCMs among married women varied from 18.2 % in a study conducted in East Wollega [23] to 65.2 % in Jinka, Ethiopia [14]. This wide inter study variation might be due to variations in settings and study population.

Yet in Sub-Saharan Africa, only 2.7 million women or fewer than one in eight (12.5 %) are currently using these contraceptive methods. Likewise, these pooled studies in Ethiopia also showed that the practice of using LAPMCs among women in the reproductive age group was on average 13.5 % (8.2–18.74 %), which is low. These low uptakes of LAPCMs might be due to the fact that LAPCMs are provider dependent methods, which are only available within the health care system (with status of “health care system access”). The health care system’s policies, structures and organization of services and the providers’ practices greatly influence client access to LAPCMs.

The meta-analysis revealed that there is a discrepancy between intentions to use LAPCMs and practice. Even though more proportions of married women had intention to use LAPCMs (42.98 %), their actual practice or utilization of LAPCMs was low (16.6 %). This implies that there might be lack of accessibility and availability of the LAPCMs in the lowest service delivery level near to the residential area of the women. In Sub Saharan Africa, the use of LAPCMs remains relatively low and LAPCMs are sometimes the missing component of national FP programs [3]. A great majority of the health facilities in Ethiopia offer oral pills (98.8 %) and injectables (98.8 %) [6].

### Strengths and limitations

The use of multiple data base for the researching of articles (both manual and electronic search), abstraction of information uniformly using a predetermined and pretested standard format by two independent reviewers can be taken as strength of this study. There are potential limitations to this study. The analysis was based on limited studies conducted in subjects with different socio-cultural and economical characteristics which might have effect on women intention to use LAPCMs and their utilization. Even though it incorporates 16 cross sectional studies from different part of the country, still the representativeness of the population is not as such strong.

## Conclusions

The meta-analysis revealed that the intention of women to use LAPCMs is generally good but the utilization of LAPCMs by women is low. It is recommended that LAPCMs must be made available and accessible for those women who are in need of it at the lower health service delivery level. In addition, client-provider interaction should be strengthened through empowering clients and having skilled, motivated service providers with appropriately staffed, managed and functioning service sites.

## Abbreviations

AOR, adjusted odd ratio; CI, confidence interval; EDHS, Ethiopian Demographic Health Survey; FP, family planning; IUCD, intrauterine contraceptive device; LAPCMs, long acting permanent contraceptive methods

## References

[1] Bekele D, Fantahun M, Gutema K, Getachew H, Lambiyo T, Yitayal M (2003). Family Planning Module, Ethiopian Health Center Team Hawassa University, USAID.

[2] Creanga AA, Gillespie D, Karklins S, Tsui AO (2011). Low use of contraception among poor women in Africa: an equity issue. Bull World Health Organ.

[3] Contraceptive security brief hormonal implants. Availableat: URL: http://deliver.jsi.com/dlvr_content/.../logisticsbriefs/CSBrie_HormImpl.pdf. Accessed 20 Sept 2014.

[4] Peterson HB, Curtis KM. Long acting methods of contraception. N Engl J Med 2005;353:2169-75. 10.1056/NEJMcp044148 16291986

[5] Bradley S, Croft T, Rutstein S (2011). The Impact of Contraceptive Failure on Unintended Births and Induced Abortions: Estimates and Strategies for Reduction. DHS Analytical Studies No. 22.

[6] UNFPA, Federal Democratic Republic of Ethiopia Ministry of Health. National Survey on Availability of Modern Contraceptives and Essential Life Saving Maternal/RH in Service Delivery Points in Ethiopia. 2010.

[7] Central Statistical Agency, mini Ethiopia Demographic and Health Survey 2014, Central Statistical Agency, Addis Ababa, Ethiopia: ICF International, Calverton, Md, USA, 2014.

[8] Takele A, Degu G, Yitayal M (2012). Demand for long acting and permanent methods of contraceptives and factors for non-use among married women of Goba Town, Bale Zone, South East Ethiopia. Reprod Health.

[9] Mekonnen W, Worku A (2011). Determinants of low family planning use and high unmet need in Butajira District, South Central Ethiopia. Reprod Health.

[10] Tarekegn, Lieberman and Giedraitis. Determinants of maternal health service utilization in Ethiopia: analysis of the 2011 Ethiopian Demographic and Health Survey BMC Pregnancy and Childbirth 2014;14:161 10.1186/1471-2393-14-161 PMC4022978 24886529

[11] Alemayehu M, Belachew T, Tilahun T (2012). Factors associated with utilization of long acting and permanent contraceptive methods among married women of reproductive age in Mekelle town, Tigray region north Ethiopia. BMC Pregnancy Childbirth.

[12] Federal Democratic Republic of Ethiopia Ministry of Health (2006). National Reproductive Heath Strategy 2006 – 2015.

[13] Federal Democratic Republic of Ethiopia Ministry Of Health: the 2011 – 2015 Health Sector Development Programme IV, 2010, Addis Ababa Ethiopia

[14] Mekonnen G, Enquselassie F, Tesfaye G, Semahegn A. Prevalence and factors affecting use of long acting and permanent contraceptive methods in Jinka town, Southern Ethiopia. The Pan African Medical Journal. 2014;18:98. doi:10.11604/pamj.2014.18.98.3421. 10.11604/pamj.2014.18.98.3421 PMC4232023 25404960

[15] Bulto GA (2014). Demand for long acting and permanent contraceptive methods and associated factors among married women of reproductive age group in Debre Markos Town, North West Ethiopia. BMC Women’s Health.

[16] Tania B, Meca JS, Martinez FM (2006). Assessing heterogeneity in meta-analysis. Q statistic or I2 index. Psychol Methods.

[17] Higgins JP, Thopson SG, Deeks JJ, Altman DG (2003). Measuring inconsistency in meta-analysis. BMJ.

[18] Meskele M, Mekonnen W (2014). Factors affecting women’s intention to use long acting and permanent contraceptive methods in Wolaita Zone, Southern Ethiopia: a cross-sectional study. BMC Women’s Health.

[19] Gebremariam A, Addissie A (2014). Intention to use long acting and permanent contraceptive methods and factors affecting it among married women in Adigrat town, Tigray, Northern Ethiopia. Reprod Health.

[20] Dashe N et al: Assessment of factors affecting women’s intention to use long acting and permanent contraceptive methods among family planning clients in Ambo town, Oromia National Regional state, Ethiopia. Available at: http://www.etpha.org/publications/abstracts-proceedings.html. Accessed on 20 March 2016.

[21] Central Statistical Agency [Ethiopia] and ICF International: Ethiopia Demographic and Health Survey 2011. Addis Ababa, Ethiopia and Calverton, Maryland, USA: Central Statistical Agency and ICF International; 2012.

[22] Haile A (2009). Demand for long acting and permanent contraceptive methods and associated factors among family planning service users, East Shoa Zone, Batu town, Ethiopia. Ethiop Med J.

[23] Tekelab T, Sufa A, Wirtu D (2015). Factors affecting intention to use long acting and permanent contraceptive methods among married women eproductive age groups in Western Ethiopia: a community based cross sectional study. Fam Med Med Sci Res.

[24] Alemu S, Tekelab and Desalegn W. Determinants of long acting and permanent contraceptive methods utilization among married women of reproductive age groups in western Ethiopia: Pan Afri Med J. 2015;21:246 doi:10.11604/pamj.2015.21.246.5835. 10.11604/pamj.2015.21.246.5835 PMC4607985 26523185

[25] Waleglign Geta. Utilization of LAPCMs and associated factors among married women in Bombe districtSithern Ethipoa. 2015. Available at: URL http://www.etpha.org/publications/abstracts-proceedings.html. Accessed on 20 March 2016.

[26] Haregewoyn Kerebh, Eskeziaw Agdew. Long acting reversible family planning utilizationand associated factors among reproductive womenin arbamich town Southern Etiopia. 2015. Available at: http://www.etpha.org/publications/abstracts-proceedings.html. Accessed on 20 March 2016.

[27] Godana W, Wondmu F, Temesgen G, Timer G, Yesuf J et al. Utilization of long acting and permanent family planning methods among women’s visiting family planning clinic in Arba Minch hospital, Ethiopia. J Healt, Med Nur 2015;15:62-71

[28] Abajobir AA (2014). Intention to use long-acting and permanent family planning methods among married 15–49 years Women in Debremarkos Town, Northwest Ethiopia. Fam Med Med Sci Res.

